# Deep Reinforcement Learning for CT-Based Non-Invasive Prediction of SOX9 Expression in Hepatocellular Carcinoma

**DOI:** 10.3390/diagnostics15101255

**Published:** 2025-05-15

**Authors:** Minghui Liu, Yi Wei, Tianshu Xie, Meiyi Yang, Xuan Cheng, Lifeng Xu, Qian Li, Feng Che, Qing Xu, Bin Song, Ming Liu

**Affiliations:** 1 School of Computer Science and Engineering, University of Electronic Science and Technology of China, Chengdu 611731, China; minghuiliu@csj.uestc.edu.cn (M.L.); tianshuxie@std.uestc.edu.cn (T.X.); meiyiyang@std.uestc.edu.cn (M.Y.); cs_xuancheng@std.uestc.edu.cn (X.C.); 2 Yangtze Delta Region Institute (Quzhou), University of Electronic Science and Technology of China, Quzhou 324003, China; 3 Department of Radiology, West China Hospital, Sichuan University, Chengdu 610041, China; drweiyi057@163.com (Y.W.); lisueradiology@163.com (Q.L.); chefeng2020@163.com (F.C.); 4 Department of Medical Laboratory Science, The Quzhou Affiliated Hospital of Wenzhou Medical University, Quzhou People’s Hospital, Quzhou 324000, China; qz1109@wmu.edu.cn; 5 Institute of Clinical Pathology, West China Hospital, Sichuan University, Chengdu 610041, China; 2019324070001@stu.scu.edu.cn; 6 Department of Radiology, Sanya People’s Hospital, Sanya 572000, China

**Keywords:** SOX9, hepatocellular carcinoma, CT images, non-invasive prediction, deep learning, reinforcement learning

## Abstract

**Background:** The transcription factor SOX9 plays a critical role in various diseases, including hepatocellular carcinoma (HCC), and has been implicated in resistance to sorafenib treatment. Accurate assessment of SOX9 expression is important for guiding personalized therapy in HCC patients; however, a reliable non-invasive method for evaluating SOX9 status remains lacking. This study aims to develop a deep learning (DL) model capable of preoperatively and non-invasively predicting SOX9 expression from CT images in HCC patients. **Methods:** We retrospectively analyzed a dataset comprising 4011 CT images from 101 HCC patients who underwent surgical resection followed by sorafenib therapy at West China Hospital, Sichuan University. A deep reinforcement learning (DRL) approach was proposed to enhance prediction accuracy by identifying and focusing on image regions highly correlated with SOX9 expression, thereby reducing the impact of background noise. **Results:** Our DRL-based model achieved an area under the curve (AUC) of 91.00% (95% confidence interval: 88.64–93.15%), outperforming conventional DL methods by over 10%. Furthermore, survival analysis revealed that patients with SOX9-positive tumors had significantly shorter recurrence-free survival (RFS) and overall survival (OS) compared to SOX9-negative patients, highlighting the prognostic value of SOX9 status. **Conclusions:** This study demonstrates that a DRL-enhanced DL model can accurately and non-invasively predict SOX9 expression in HCC patients using preoperative CT images. These findings support the clinical utility of imaging-based SOX9 assessment in informing treatment strategies and prognostic evaluation for patients with advanced HCC.

## 1. Introduction

Hepatocellular carcinoma is one of the most common cancers and the third leading cause of cancer-related death worldwide [1]. It is also a highly aggressive liver tumor containing cancer stem cells (CSCs), which contribute to tumor growth, resistance to conventional therapies, and the promotion of tumor recurrence [2]. While liver resection is considered the first-line treatment for patients with early-stage HCC and well-preserved liver function, it is not always suitable for those with advanced-stage HCC. For these patients, systemic therapies are recommended as an alternative. Sorafenib, a multikinase inhibitor, is the only FDA-approved first-line therapy recommended by the American Association for the Study of Liver Diseases (AASLD) and has been shown to potentially extend the lives of patients with advanced HCC by 2 to 3 months [3,4]. However, the high level of resistance in HCC patients significantly impacts the clinical efficacy of Sorafenib, often leading to treatment failure [4]. Therefore, identifying patients who may benefit from sorafenib treatment without developing resistance is crucial for making individualized treatment decisions in HCC.

The transcription factor SRY box 9 (SOX9) is a key regulator involved in various diseases, including cancers. It plays a crucial role in physiological and pathological processes, such as the cell growth, apoptosis, invasion, and metastasis of tumor cells [5,6]. In hepatocellular carcinoma, SOX9 is essential for the self-renewal and proliferation of liver cancer stem cells, which contribute to tumor progression and drug resistance. Additionally, SOX9 has been identified as a potential CSC marker and an independent prognostic factor for HCC [2,7,8]. Recent studies have shown that SOX9 is overexpressed in many solid tumors, including HCC [7,9,10]. Furthermore, a high SOX9 expression correlates with tumor aggressiveness in liver cancer and enhances sorafenib resistance by modulating the expression of ATP-binding cassette sub-family G member 2 (ABCG2) [11]. Therefore, predicting SOX9 expression in advanced HCC could help identify patients at risk of sorafenib resistance, enabling timely alternative therapies. However, SOX9 status is typically assessed through immunohistochemistry, which requires invasive tumor samples to be obtained via surgery or biopsy. This procedure may introduce sampling bias and increase patient morbidity. Thus, a non-invasive and efficient method for assessing SOX9 expression is urgently needed to enable personalized treatment strategies for HCC patients. This has motivated us to propose a non-invasive method for predicting SOX9 expression, aiming to reduce the physical burden and economic strain on patients.

Recently, deep learning has been widely applied in various fields, including computer vision [12], natural language processing [13], DNA sequence analysis [14], and medical image classification [15]. Computed tomography (CT) images, a well-known form of medical imaging, contain high-dimensional features that are closely related to a tumor’s microenvironment and molecular status. Many studies have applied DL models to CT images to predict disease grade, gene expression, and immune checkpoint status, including the expression of SOX9 [16,17,18]. However, not all regions of a CT image are equally informative about a patient’s SOX9 status. While DL models excel at handling noisy data and capturing high-dimensional features, it remains a challenging task for neural networks to independently identify regions relevant to SOX9 expression.

The novelty of this study lies in incorporating reinforcement learning into a deep learning model to guide the model in identifying the most relevant regions in CT images, thereby reducing the effect of background noise on the predictive model and enhancing its performance. RL is an advanced technique that trains an agent to take actions in an environment to maximize its cumulative rewards. By optimizing a series of strategies, the model can adjust key parameters to improve its performance [19,20,21]. In our work, RL helps identify the regions in CT images that are most indicative of SOX9 expression status. To the best of our knowledge, this is the first study to utilize a deep learning model enhanced by reinforcement learning to predict SOX9 expression status from CT images. This method not only provides a potential high-precision non-invasive approach for gene expression prediction but also introduces a technique that enhances regions of interest through deep reinforcement learning while effectively mitigating background noise interference, significantly improving the performance of the predictive model.

In conclusion, we have developed and validated a non-invasive deep learning model that can predict SOX9 expression status in patients with advanced HCC using only preoperative contrast-enhanced CT images. We also examined the relationship between SOX9 expression and prognosis in HCC patients treated with sorafenib after surgery. Our experiments demonstrate that the proposed model outperforms previous methods by effectively focusing on relevant regions, indicating its potential to inform personalized treatment strategies for patients with advanced HCC. Furthermore, our findings suggest a strong correlation between SOX9 expression status and deep features extracted from medical imaging, opening up new avenues for research in HCC treatment.

## 2. Predictive Model

**Figure 1:**
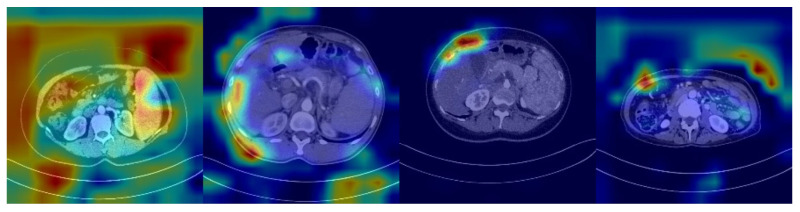
Heatmap from a ResNet-based model showing a SOX9-positive prediction, with darker regions indicating more attention from the model.

The features potentially seen in CT images may reflect the expression of many genes in HCC patients, including SOX9. Unlike radiomic analyses, deep learning models can automatically identify features in CT images related to SOX9 expression, rather than being limited to known, predefined biomarkers. This enables deep learning models to learn and predict more holistically. However, despite the significant improvement in their classification performance compared to that of traditional machine learning methods, deep learning models are still inevitably affected by noise, especially from features in low-correlation regions. As shown in Figure 1, which presents the heatmap of a SOX9-positive prediction based on ResNet, deeper colors indicate regions receiving higher attention from the model. However, the model tends to focus on noisy areas, which significantly hampers its performance.

**Figure 2:**
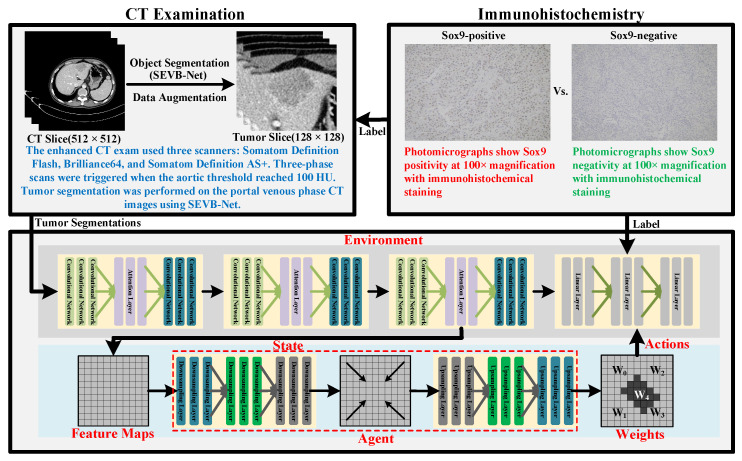
Diagram illustrating the workflow of the proposed RL-based prediction model.

In this study, we developed a novel model that uses reinforcement learning (RL) to guide its attention toward regions closely associated with SOX9 expression while using only preoperative contrast-enhanced CT images to predict SOX9 status in patients with advanced HCC. Compared to traditional methods, our model can automatically identify and strengthen key regions while minimizing interference from noisy areas, thereby significantly improving its prediction accuracy and robustness. This model not only effectively enhances its classification performance but also provides more precise support for personalized treatment planning. The workflow of the proposed method is illustrated in Figure 2. The top two boxes demonstrate the CT examination and immunohistochemistry procedures considered by our model. The box at the bottom outlines the framework of the proposed model. All components within the model are parameterized by neural networks, with layers that remain trainable throughout the entire training process. The upper half of the framework corresponds to the classification model, while the lower half represents the proposed reinforcement learning method.

We redefine the prediction task as a binary classification problem, the problem of distinguishing between SOX9-positive and SOX9-negative cases, to enhance the model’s expressiveness in this task. To improve its classification performance and minimize interference from background noise, the proposed framework consists of two key components: a classification model and a reinforcement learning model. The classification model is built upon a residual network [12], with an attention layer added to enhance its classification performance and facilitate long-range feature modeling. The reinforcement learning model consists of a generator, comprising an encoder and a decoder, that acts as an RL agent. By generating a weight matrix, this procedure enables the model to focus selectively on high-weighted regions of interest, effectively filtering out irrelevant areas.

### 2.1. Classification Model

The classification model is based on an enhanced residual network, which includes an attention layer to effectively capture long-range feature dependencies. The attention layer incorporates a self-attention mechanism [13,22,23], enabling the model to learn global dependencies across the input features. By computing the correlations between a Query (Q), Key (K), and Value (V), attention weights are generated to dynamically adjust the importance of features, thus enhancing the model’s ability to understand and utilize global information. This attention layer is seamlessly integrated into the residual blocks in a modular manner, thereby ensuring more efficient feature propagation throughout the network. This process can be mathematically described as follows:

(1) $$\left\{\begin{array}{cc} Q & =XW_{Q}\\ K & =XW_{K}\\ V & =XW_{V}\\ A & =softmax(\frac{QK^{T}}{\sqrt{d_{k}}}) \end{array}\right.$$

Here, *Q*, *K*, and *V* are obtained by linearly transforming the input features *X* and *A* represents the attention weights.

The cross-entropy loss function [24] has been widely used in the training of classification models to optimize their performance, as shown in Equation (2). By measuring the difference between the predicted results and the true labels, it penalizes predictions that deviate significantly from the true values. This approach guides the model to continuously adjust its parameters, minimizing its error and improving the accuracy of its gene expression prediction. In this study, we applied the softmax transformation to the standard function to obtain the probability distribution of the results, thereby enhancing both the interpretability and readability of the outcomes.

(2) $$L=-\log(\frac{\exp\left(w\times (\nu ⊕\mu )[\lambda ]\right)}{\sum_{i}\exp\left(w\times (\nu ⊕\mu [i]\right)})$$

where λ represents the experimentally validated SOX9 gene expression in patients, while *i* denotes the different categories possible.

### 2.2. Reinforcement Learning Model

The training and updating of the reinforcement learning (RL) model can be viewed as an interaction between the generator and the classification model. From the generator’s perspective, the feature map output from the final residual block in the classification model is treated as the state which is then fed into the generator as a request. The generator responds by generating a new weight distribution and feeds it back to the classification model as an action. This process continues iteratively, with the generator making new decisions based on received signals, to adaptively generate weight distributions that focus on the CT regions most indicative of SOX9 expression. In this process, the generator functions as the RL agent. Finally, by modeling the classification model as the environment and classification accuracy as the reward, the overall process can be framed as a reinforcement learning problem. Let M=<S,A,T,r,π> represent the agent’s decision-making process, where *S* denotes the observation space, *A* is the action space, and *T* is the transition function that determines the next state based on the current state–action pair. The goal is to learn a policy π that maximizes the expected cumulative reward. The reward function *r*, defined as having a benchmark value of 0.9, evaluates the effectiveness of an action by comparing the prediction’s accuracy to this benchmark. If the prediction’s accuracy exceeds 0.9, a positive reward is given, signaling a desirable outcome. Conversely, if the accuracy falls below 0.9, a negative reward is assigned, indicating that the action did not meet the performance threshold. This feedback mechanism guides the agent toward actions that enhance prediction accuracy.

Proximal Policy Optimization (PPO) [25] is a widely used policy gradient method designed for reinforcement learning. It enables data sampling through interactions with the RL environment and optimizes a surrogate objective function using a stochastic gradient descent. In this study, agent training follows the Proximal Policy Optimization-Clip (PPO-Clip) approach, which introduces a similarity constraint that limits policy deviation during importance sampling. The similarity constraint is summarized as follows:

(3) $$\begin{array}{c} J_{PPO-Clip}^{\theta^{k}}\left(\theta \right)\approx \sum_{s_{t},a_{t}}\min(\frac{p_{\theta }\left(a_{t}∥s_{t}\right)}{p_{\theta }^{k}\left(a_{t}∥s_{t}\right)}A^{\theta^{k}}\left(s_{t},a_{t}\right),\\ clip\left(\frac{p_{\theta }\left(a_{t}∥s_{t}\right)}{p_{\theta }^{k}\left(a_{t}∥s_{t}\right)},1-\epsilon ,1+\epsilon \right)A^{\theta^{k}}\left(s_{t},a_{t}\right)) \end{array}$$

Let $a_{t}$ and $s_{t}$ denote the model’s action and state at step *t*, respectively. Here, $p_{\theta }\left(a_{t}∥s_{t}\right)$ represents the output policy, while $p_{\theta }^{k}\left(a_{t}∥s_{t}\right)$ refers to the target policy. The hyperparameter ϵ is used to measure the distance between the output policy $p_{\theta }\left(a_{t}∥s_{t}\right)$ and $p_{\theta }^{k}\left(a_{t}∥s_{t}\right)$.

### 2.3. Alternating Training

The classification model and the reinforcement learning model are trained alternately to ensure their stable convergence. While training the classification model, its parameters are updated using the cross-entropy loss, with the reinforcement learning model remaining fixed. Conversely, when training the reinforcement learning model, only its own parameters are updated. This alternating training strategy ensures that both models influence each other while maintaining a certain level of independence. As a result, this approach helps stabilize the overall training dynamics of the model, allowing each model to learn effectively without overwhelming the other, thereby improving the overall performance of the system. Algorithm 1 provides a detailed description of this process in pseudocode.
**Algorithm** **1** Pseudocode of the alternating training.  1:**Inputs:** Training data data $=(x_{i},y_{i})$  2:**Outputs:** the Classification Model *M* and the Generator *G*  3:Initialize parameters of both models  4:**for** each epoch in epochs **do**  5:      **for** each batch data*_i_* in data **do**  6:            Freeze the parameters of the generator  7:            Calculate the loss value using the cross-entropy loss(Equation (2))  8:            Update the parameters of the classification model  9:      **end for**10:      **for** each batch data*_i_* in data **do**11:            Freeze the parameters of the classification model12:            Calculate the reward13:            Update the parameters of the Proximal Policy Optimization-Clip approach (Equation (3))14:      **end for**15:**end for**

## 3. Patient Cohort and Data Collection

**Figure 3:**
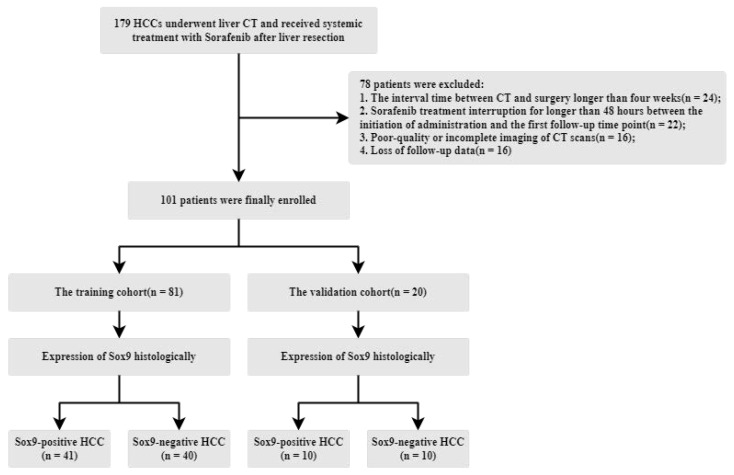
Illustration of the patient recruitment process, data division, and the inclusion/exclusion criteria.

The Institutional Review Board of West China Hospital approved this retrospective study, with informed consent being waived due to its retrospective nature. From an initial cohort of 179 patients with histologically confirmed hepatocellular carcinoma (HCC) who underwent systemic sorafenib treatment (800 mg daily, administered in the form of 400 mg twice per day) following surgery between July 2011 and June 2019, a total of 101 patients were included. The exclusion criteria were as follows: (1) the interval between CT and surgery exceeded four weeks, (2) poor-quality or incomplete CT imaging, (3) sorafenib treatment was interrupted for more than 48 h between the start of its administration and the first follow-up, and (4) a lack of follow-up data. The patient recruitment and data allocation processes are illustrated in Figure 3. A total of 78 patients were excluded due to data reliability issues. The remaining patients were then split into training and validation cohorts. To ensure an equal evaluation, the number of SOX9-positive and -negative patients in the validation cohort was balanced.

The median age of the enrolled patients was 51 years (IQR: 42–60). In total, 90 were male (89.11%) and 11 were female (10.89%). The median overall survival time was 29.23 months, while the median recurrence time was 13.27 months. The corresponding interquartile ranges for survival and recurrence were 14.56–47.7 months and 4.77–24.7 months, respectively. An immunohistochemistry analysis showed that 51 patients were SOX9-positive, with a median age of 52 years (IQR: 42–62), while the remaining 50 patients were SOX9-negative, with a median age of 50 years (IQR: 43–59). No significant differences were observed in age distribution between the SOX9-positive and SOX9-negative groups. Additionally, a total of 4011 high-quality CT images were collected, of which 2041 were from the SOX9-positive group, indicating that there were no significant differences in CT image sampling and screening between the two groups.

Finally, patients were randomly assigned to either the training cohort or the validation cohort using the classic hold-out strategy [26], with 20 patients in total allocated for validation. This group included 10 (50%) from the SOX9-positive group and 10 (50%) from the SOX9-negative group. The training cohort consisted of 72 males and 9 females, with a median age of 52 years (IQR: 43–61). In contrast, the validation cohort included 18 males and 2 females, with a median age of 49 years (IQR: 40–60). No significant differences were observed between the training and validation cohorts in terms of sex or age distribution. A summary of the cohorts’ characteristics is presented in Table 1. The data interpreted include PLTs (Platelets), Neutrophils, Lymphocytes, TBIL (Total Bilirubin), ALT (Alanine Aminotransferase), AST (Aspartate Aminotransferase), ALB (Albumin), GGT (γ-glutamyl transpeptidase), AFP (Alpha-Fetoprotein), and CEA (Carcinoembryonic Antigen).

### 3.1. Follow-Up Surveillance

All patients were regularly followed up with 3 to 6 months after a curative liver resection, with tests for α-fetoprotein levels and imaging examinations, such as ultrasound, CT, or MRI, conducted. During this follow-up, we recorded the time of disease-specific progression (including local recurrence or distant organ metastasis) or death. Recurrence-free survival (RFS) was defined as the time between surgery and the date of relapse. Overall survival (OS) was calculated as the interval from the surgery date to either the date of death or the most recent follow-up. Data from patients who were still alive at the last follow-up were censored.

### 3.2. Immunohistochemistry

Surgically resected specimens embedded in paraffin were sliced into 4 μm thick sections, dewaxed, hydrated, and subjected to antigen retrieval. The tissue slides were incubated overnight at 4 °C with a primary monoclonal antibody (1:200 rabbit monoclonal antibody, HuaBio, ET1611-56), followed by incubation with a secondary antibody (cat # K5007) and Dako. SOX9 staining was then performed using 3,3′-diaminobenzidine, and counterstaining was achieved with hematoxylin. Two senior pathologists, blinded to all radiological and clinical data, independently assessed the histopathological slides. They performed statistical analyses by selecting five non-overlapping and non-continuous regions to calculate the mean. SOX9-positive cells were quantified at a 400× magnification (0.0484 mm^2^), and SOX9 expression was determined based on the proportion of SOX9-positive tumor cells. A threshold of 5% for the ratio of SOX9-positive tumor cells to total tumor cells was set, with samples exceeding this threshold considered SOX9-positive and those below it considered negative. If the results showed a variation within 5% of the threshold, re-assessments were conducted until a consensus was reached.

### 3.3. CT Image Acquisition and Processing

Slices from contrast-enhanced CT scans were obtained using three types of multi-detector CT (MDCT) scanners: the Somatom Definition Flash (used for 45% of the included patients), the Brilliance64 (used for 20% of the included patients), and the Somatom Definition AS+. The high-resolution scanning protocol was as follows: a tube voltage of 90–120 kVp, amperage of 200–20 mA, rotation time of 0.5–0.75 s, pitch of 0.8–1.0, and slice thickness of 2 mm. Intravenous non-ionic contrast material (Omnipaque, 350 mg/mL) at a dosage of 1.5–2.0 mL/kg, supplied by GE Healthcare Chicago IL, was administered via a power injector at a rate of 3 mL/s. Three-phase scans were conducted when the trigger threshold of the aorta reached 100 HU.

All CT images were retrospectively reviewed by two radiologists with over eight years of experience in liver imaging who were blinded to the clinicopathological data. Tumor segmentation was performed on the initial portal venous phase (PVP) CT images using SEVB-Net, a modified version of V-Net [27] developed by United Imaging Intelligence [28]. Unlike U-Net [29], SEVB-Net enables three-dimensional segmentation, rather than just working with two-dimensional images. The Dice score for this segmentation task was 0.855. After the network segmented all tumors, the radiologists reviewed and verified the results to ensure accuracy. If the network’s segmentation results were inaccurate, the tumor contours were manually delineated using ITK-SNAP software (version 3.6).

Finally, the CT images were centered around the tumor, extending outward according to the segmentation results until the size of the image reached 128 × 128. If the tumor boundary exceeded 128, the boundary was preserved. To meet the network requirements, we resized all images to a unified scale of 128 × 128. Additionally, several data augmentation techniques were applied, including Random Rotation, Random Cropping, Random Horizontal Flipping, Random Vertical Flipping, and ColorJitter [30], to increase data diversity and improve the model’s generalization ability. To mitigate the impact of sample imbalance on model training, we performed upsampling on the less abundant samples to improve training efficiency and stabilize the dynamics of the training process.

## 4. Results

### 4.1. Overview

In this section, we first compare the performance of various deep learning models with our proposed method to comprehensively evaluate their ability to predict SOX9 expression. To evaluate their predictive performance, we employed accuracy, sensitivity, specificity, and receiver operating characteristic (ROC) curves as evaluation metrics. Next, we visualized the regions of interest highlighted by our model in the CT images to verify the effectiveness of its reinforcement learning. Finally, we used the Kaplan–Meier (KM) method to generate survival curves for overall survival and recurrence-free survival in patients with different SOX9 expression levels.

### 4.2. Experimental Setup

During the model training phase, the model was trained for 600 epochs. In each epoch, we first froze the parameters of the generator model and focused on training the classification model. This step aims to update the parameters of the classification model with the assistance of the generator model. Then, we froze the parameters of the classification model and trained the generator model instead to calculate the reward and update the generator model’s parameters. This alternating training allows for effective interaction between the model and the environment, further helping the deep learning model find the optimal areas for SOX9 prediction. The classification model has a depth of 56. Table 2 presents the core modules of the model proposed in this study, along with their hierarchical layers. The first column displays the models, the second column lists their respective layers, the third column shows their current input, and the fourth column presents their corresponding output. In terms of parameter settings, the optimizer used for the classification model is SGD, while the optimizer used for the generator model is Adam. The initial learning rate for the classification model was set to 0.1, while that for the generator model was set to 0.01, decaying by a factor of 0.1 every 100 epochs. Additionally, the batch size was set to 32, and the weight decay was set to 0.0005. During the validation phase, for each patient, the average probability of all CT images was calculated as the final probability of SOX9 expression in that patient. All deep learning experiments were implemented using Python version 3.12.8 and Pytorch version 2.5 on two NVIDIA GeForce GTX 1080Ti GPUs.

### 4.3. Predictive Performance of the Model

**Figure 4:**
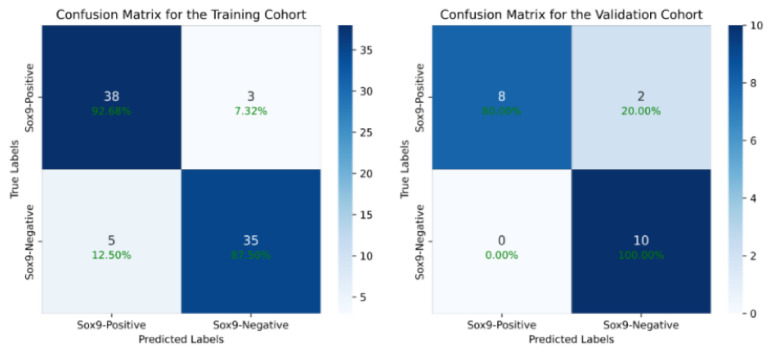
The confusion matrix of our model’s output, which shows that we have achieved a good balance between sensitivity and specificity, significantly outperforming other existing models.

We first evaluated the performance of our proposed deep learning model by comparing it with traditional models. On the training cohort, our method achieved an accuracy (ACC) of 90.12% and an area under the curve (AUC) of 94.42% (95% CI: 92.23–96.39%) for SOX9 prediction. On the validation cohort, the model attained an ACC of 90.00% and an AUC of 91.00% (95% CI: 88.64–93.15%). Table 3 presents a comparison of our method with previous deep learning models, with ROC curves used on the validation cohort. The results demonstrate that our approach outperformed all other models, achieving the highest AUC. Compared to the baseline models, our model achieved a 17.58% increase in accuracy and a 16.07% increase in AUC on the training set. On the validation set, the model achieved a 10% increase in accuracy and a 19% increase in AUC. Compared to existing models, our method improved in its ACC by at least 10% and its AUC by at least 11%, highlighting its superior predictive performance. The DeLong test is a statistical method that calculates a statistic for the difference between two AUCs, compares it with the standard normal distribution, and obtains a p-value to assess the performance difference between two or more receiver operating characteristic (ROC) curves. The experimental results show that the p-value of the difference between our model and other models is less than 0.001, indicating a significant statistical difference in the AUC of our model and the others. Figure 4 presents the confusion matrix of the results of the model proposed in this study. It can be observed that the model achieves a good balance between sensitivity and specificity, significantly outperforming other existing models. This advantage is attributed to our effective identification of key regions in the images using reinforcement learning, which further validates the effectiveness of our algorithm.

Notably, our model not only achieved a significant improvement in accuracy but also demonstrated the best performance across both the training and testing cohorts, with the smallest discrepancy seen between these cohorts. This indicates that our approach effectively mitigates environmental noise interference and accurately captures key features highly correlated with SOX9 expression, making it very suitable for providing clinical diagnostic assistance. Furthermore, compared to the original model without reinforcement learning-based feature selection, our method achieved improvements of over 7% across all evaluation metrics, with a maximum increase of up to 19%. Notably, the enhancements observed in the validation cohort exceeded 10%, further validating the superiority and advanced capabilities of our proposed model.

**Figure 5:**
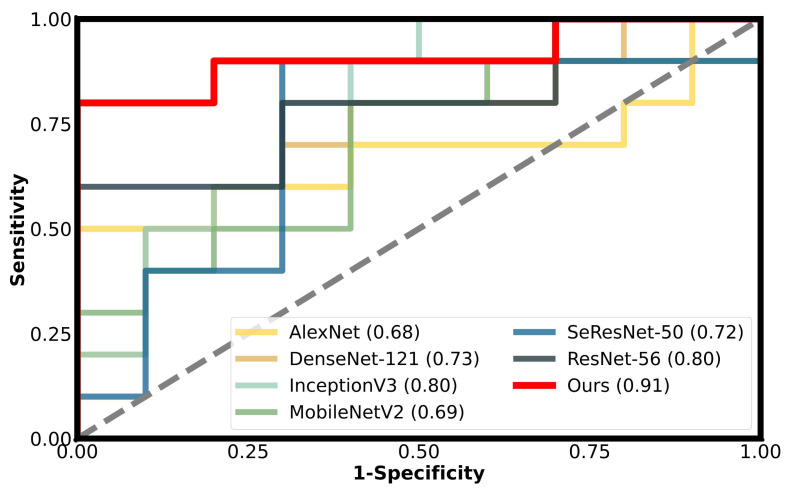
The performance of different models on the validation cohort is shown using ROC curves, with our method represented by the red solid line.

Figure 5 presents a comparison of our model with other traditional models in terms of their ROC curves, where the y-axis represents the true positive rate (TPR) of SOX9 expression, and the x-axis represents the false positive rate (FPR). The closer the curve is to the top-left corner, the better the model’s performance. The results indicate that our model achieves the best performance at almost all reference points, which strongly demonstrates the critical role of reducing background noise in enhancing the predictive performance of our model.

### 4.4. Sensitivity Analysis of Parameters

**Figure 6:**
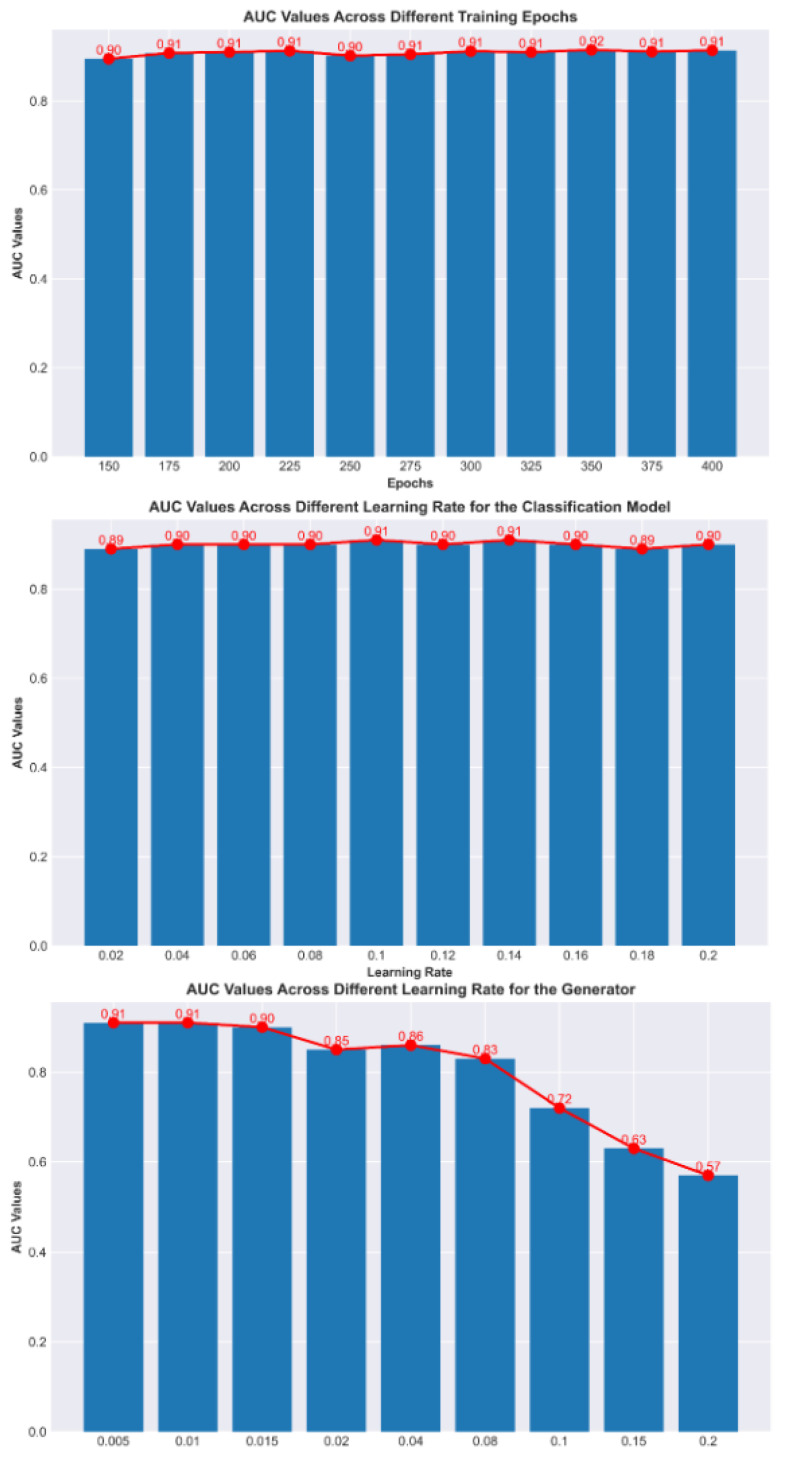
Comparison of AUC values under different parameter settings.

Figure 6 shows the impact of different training parameters on model performance. It can be observed that the number of training epochs and the initial learning rate of the classification model have minimal impact on the model’s results, indicating that our model is highly robust and can achieve an optimal performance across various parameter settings. However, the initial learning rate of the reinforcement learning has a significant effect, especially when the initial value is large.

This is because reinforcement learning can interfere with the parameters of the classification model. When the model is in its initial state, if the initial learning rate of the reinforcement learning model is too high, it may cause interference that is larger the learning capacity of the classification model, leading to instability in the training process. Moreover, the larger the initial learning rate, the more difficult the training becomes. To address this issue, either the initial learning rate of the reinforcement learning model can be reduced or a warm-start approach (such as pre-training) can be used to alleviate instability during training.

### 4.5. Interpretability of Reinforcement Learning

**Figure 7:**
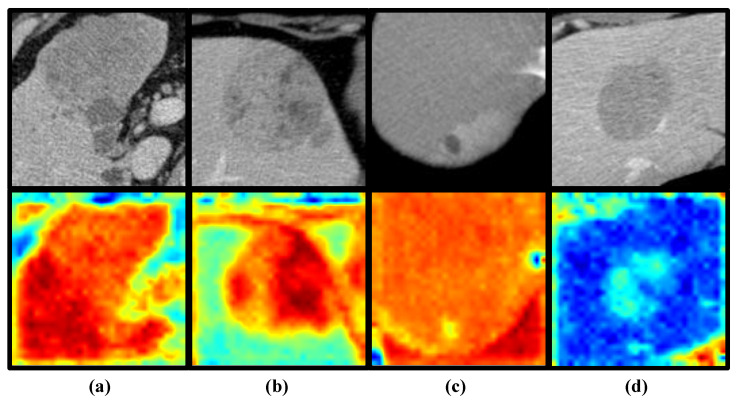
Examples of class activation maps for TP (**a**), TN (**b**), FP (**c**), and FN (**d**) samples, where green, yellow, and red represent different areas given attention by the model. The deeper the color, the higher the level of attention the model gives to that region.

To better demonstrate the impact of reinforcement learning on the model, we use class activation mapping (CAM) [34] to visualize the signs of reinforcement learning. CAM is a technique that projects the weights of the output layer back onto the convolutional feature map to guide the model’s attention to key regions of the image, enabling the visualization of the model’s focus. Figure 7 shows the attention activation maps generated for true positive (TP), true negative (TN), false positive (FP), and false negative (FN) samples, with panels a, b, c, and d representing the results for each of these four sample types. The most important features of the TP and TN samples (panel a and panel b) were consistently located around the tumor, whereas for FP and FN samples (panel c and panel d) the model’s attention was more centered on the background regions. This suggests that focusing the model’s attention on the tumor and its surrounding areas helps improve its prediction of SOX9 expression in liver cancer patients. On the other hand, it also indicates that background noise significantly impacts the model’s performance in predicting SOX9 expression. Excessive background interference can lead to inaccurate predictions, further emphasizing the crucial role of reducing background noise in enhancing the model’s accuracy. The visualization results fully validate the motivation and effectiveness of the method proposed in this paper. By avoiding the interference of background noise, our model is not only better able to identify key features and focus on the region of the tumor, significantly improving prediction accuracy; it is also able to enhance its robustness in complex environments.

### 4.6. The Association Between SOX9 Expression and Prognosis

**Figure 8:**
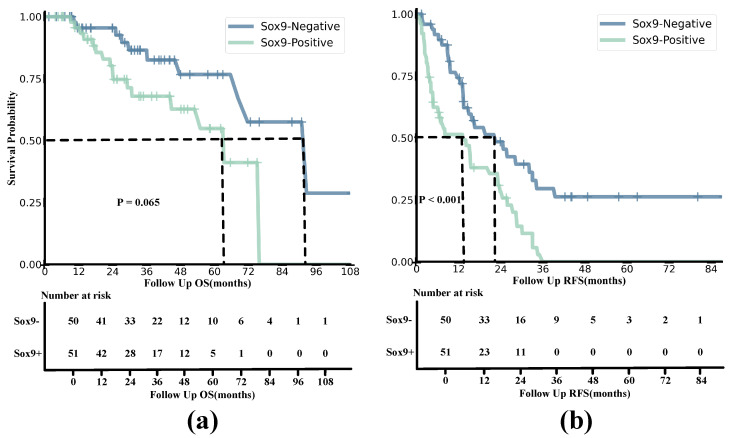
Kaplan−Meier analyses of OS (**a**) and RFS (**b**) based on positive and negative SOX9 expression in HCC patients from both the training and validation cohorts. The survival probability is shown on the *y*–axis, and the follow-up time is presented on the *x*–axis.

The curves for RFS and OS indicate that SOX9 expression is closely associated with the prognosis of HCC patients, with the Kaplan–Meier (KM) method able to describe survival outcomes, as shown in Figure 8. The median OS for the SOX9-positive and -negative groups was 25.57 and 32.27, respectively, with corresponding interquartile ranges (IQRs) of 13.87–44.8 and 14.56–48.0. On the other hand, the SOX9-positive group had a median RFS of 7.03 and an IQR of 3.4–23.5, while the SOX9-negative group had a median RFS of 13.98 and an IQR of 9.1–30.53. The estimated 1-year RFS rates for the SOX9-positive and -negative groups were 52.94% and 76.00%, respectively, and the 2-year OS rates were 82.35% and 96.00%. The Kaplan–Meier analysis results for OS and RFS are presented in the following figures.

#### Overall Survival

The results demonstrate that the survival probability and OS of SOX9-positive patients were significantly lower than those of SOX9-negative patients. The survival rate of SOX9-positive patients decreased relatively slowly compared to that of the SOX9-negative group. According to the Log-Rank Test, the $\chi^{2}$ value of OS being related to SOX9 status was 3.400, with a df of 1.00 and a *p*-value of 0.065.

### 4.7. Recurrence-Free Survival

Similarly to the OS curves, the results indicated that the survival probability and RFS of SOX9-negative patients were consistently higher than those in the SOX9-positive group. Moreover, the survival probability of the SOX9-positive group dropped to zero in a short amount of time, while the trend for the SOX9-negative group remained relatively smooth. The results of the Log-Rank Test were as follows: $\chi^{2}$ = 13.153; df = 1; and p<0.001. This indicates that the model rejected the null hypothesis, and it is meaningful for construction.

## 5. Discussion

Some studies have started exploring the feasibility of using machine learning or deep learning techniques to predict gene expression in cancer patients and have made some progress. Suleyman et al. [35] employed five machine learning techniques, Random Forest, Support Vector Machine (SVM), Naive Bayes, C4.5, and K-Nearest Neighbors, to analyze somatic mutation data in breast cancer patients. When using the Random Forest method, the study achieved an accuracy of 0.70. Bhalla et al. [36] applied SVM- and Random Forest (RF)-based models to 523 cases of clear cell renal cell carcinoma (ccRCC). The main goal of the study was to identify the minimum number of biomarker genes that could effectively distinguish between early-stage and late-stage ccRCC, enabling accurate cancer staging, and this was accomplished with a final accuracy of 70.19%. Matsubara et al. [37] used convolutional neural networks (CNNs) combined with spectral clustering information to classify lung cancer using protein interaction network data and gene expression data from 639 samples. The accuracy, recall, precision, and specificity achieved in the study were 0.81, 0.88, 0.78, and 0.74, respectively. Guillermo et al. [38] proposed the combination of CNN and Transfer Learning (TL) for lung cancer prediction. Their study utilized data from TCGA, which contains 33 different types of cancer, with a focus on testing the lung cancer dataset, and achieved an accuracy of 68%. It can be observed that due to the limitations of these method’s effectiveness, the performance of existing methods has reached a bottleneck, with a maximum accuracy of only 0.81. This is not only because the feature extraction process of existing methods heavily relies on expert experience, but also because these methods fail to effectively extract deep features, and particularly the relationships between features. Therefore, research focused on gene prediction needs to further improve the performance of model feature extraction.

On the other hand, current gene prediction technologies primarily rely on genetic sequence data, overlooking the potential of medical imaging (e.g., CT scans) in disease prediction. Genetic sequences provide valuable genetic information, but this approach has certain limitations. First, relying solely on genetic sequences for predictions may fail to capture the full complexity of a disease, particularly in terms of the spatial heterogeneity of tumors and the influence of the tumor microenvironment. Medical imaging, on the other hand, can directly present the tumor’s shape and size and changes in the surrounding tissues, offering more timely and dynamic information. Second, genetic sequencing usually requires additional testing steps, increasing the financial and time burdens on patients, whereas medical imaging is relatively more straightforward and can reflect the disease’s progression in real time. Finally, the accuracy of genetic sequence predictions is often affected by factors like genetic heterogeneity, making it difficult to meet clinical needs, while medical imaging provides more intuitive clinical evidence. Therefore, compared to relying solely on genetic sequences, medical imaging can be more effective in supporting disease diagnosis and prediction, especially in the study of complex diseases like cancer.

Recently, as an important transcription protein, SOX9 has attract a lot of attention due to the relationships between SOX9 and cancer progression and drug resistance [2,7,8]. In terms of HCC, a recent study has proven that high SOX9 expression levels indicate tumor aggressiveness in liver cancer and enhance sorafenib resistance by modulating ATP binding cassette sub-family G member 2(ABCG2) expression [11]. Furthermore, sorafenib is a first-line systematic treatment option for patients with advanced HCC. Therefore, identifying the SOX9 status of patients can help to determine the risk of sorafenib resistance, which is essential for the construction of personalized treatment strategies, including the choice to use alternative therapies like ICIs. However, IHC is the only method currently available for detecting SOX9 expression, and it requires invasive biopsy or surgery. This invasive strategy may bring the risk of sampling bias and morbidities. Therefore, demonstrating the association between SOX9 and medical imaging could open up new research opportunities in medical imaging analysis while also providing a non-invasive, preoperative SOX9 detection strategy that supports precision treatment for HCC.

Representations from CT images were found to be informative for disease grading, gene expression, and assessing the status of the immune checkpoint pathway. A series of previous studies [39,40,41,42] utilized radiomics combined with qualitative and quantitative analyses to predict gene or phosphorylation expression through manual feature extraction. Although these proof-of-principle studies demonstrated that radiomics features provide a comprehensive overview of tumor pathological status, feature extraction relying on human expertise still faces challenges such as feature bias, information loss, and reproducibility. Convolutional neural networks, as deep learning techniques, offer significant potential for feature extraction and diagnosis and have led to breakthroughs in many domains [12,13,14,15]. They can naturally integrate various features and classifiers in an end-to-end, multi-layer fashion, and the levels of these features can be enriched by increasing the depth [12]. This technology has been applied to prediction tasks based on medical images [16,17,18]. Although CNNs have shown promising performances in feature extraction and prediction tasks, they still struggle to recognize the actual discriminative regions relevant to their prediction target. Specifically, in our task of predicting SOX9 expression, not every region of a CT image is relevant, but only certain parts of it, which suggests that a model’s diagnostic performance can be improved by enhancing those specific regions. However, it remains a challenging task to help CNNs identify these regions.

Recently, several studies have attempted to integrate deep learning with reinforcement learning to enhance the feature extraction capabilities of convolutional neural networks, achieving promising progress. Joseph Stember et al. [43] used reinforcement learning to classify 2D brain MRI images, using a very small training dataset, and achieved remarkable accuracy. However, the dataset in their study was quite limited, making it insufficient to validate the model’s generalization ability. Moreover, the study employed reinforcement learning in a multi-step image classification strategy aimed at building an end-to-end learning process, but it did not help the model selectively extract features from regions of interest. Similarly, Emma Slade et al. [44] proposed an active learning framework based on deep reinforcement learning to optimize the efficiency of medical image classification by selecting a subset of images that maximally enhance model performance. In addition, Mingyuan Jiu et al. [45] introduced an adaptive active learning method that combines deep reinforcement learning with active learning. By leveraging the Deep Deterministic Policy Gradient (DDPG) algorithm, their framework dynamically optimizes sample selection strategies across different learning environments, thereby improving model performance. In both studies, reinforcement learning was used for sample selection to enhance the representativeness of the samples used in active learning.

Although some studies have explored integrating deep learning with reinforcement learning for computer vision tasks, particularly in medical image processing and analysis, current research mainly focuses on using reinforcement learning for multi-step feature extraction and active learning decision-making. There remains a lack of research into using reinforcement learning to help models focus on regions of interest, thus reducing the impact of background noise. Experimental results have shown that background noise significantly influences the prediction of SOX9 expression in HCC patients when using CT images. Therefore, how to utilize reinforcement learning to enhance deep learning models’ ability to extract key features while effectively avoiding interference from background noise is the core focus of this study.

In this retrospective study, we developed and validated a reinforcement learning (RL)-based deep learning (DL) model that can non-invasively identify the SOX9 status of hepatocellular carcinoma (HCC) patients preoperatively, providing support for personalized treatment. A total of 101 hepatocellular carcinoma (HCC) patients, all histologically confirmed, were enrolled from West China Hospital between 2011 and 2019. These patients were subsequently divided into two groups: a training cohort and a validation cohort. The training cohort was used to develop and optimize the model, while the validation cohort was used to assess its performance and generalizability. Experimental results showed that our model achieved AUCs of 94.42% (95% CI, 92.23–96.39%) and 91.00% (95% CI, 88.64–93.15%) for the two cohorts. The results demonstrate that using deep reinforcement learning to extract latent features from CT images for the non-invasive prediction of SOX9 expression in HCC patients holds significant potential for clinical application. The model not only meets the standards required for clinical research but also strikes a good balance between sensitivity and specificity, with strong generalization capability. Compared to existing deep learning models, our algorithm shows a significantly superior performance, which is attributed to the use of reinforcement learning to focus on extracting and learning features from regions of interest, effectively mitigating the impact of background noise on the model’s performance. The use of class activation Maps to interpret the results further supported this conclusion. As shown in Figure 7, the correctly predicted samples contained regions with higher activation values that were consistently concentrated around the tumor. In contrast, the activation regions in misclassified samples were predominantly in the background. Additionally, we observed another interesting phenomenon: in the SOX9-positive group, the regions with the highest activation values were often located in the tumor and in peritumoral areas, whereas in the SOX9-negative group, the activated regions were limited to the tumor alone. Although this phenomenon was observed in only a few cases, it holds significant research value for future studies.

Previous studies have established that SOX9 is a significant biological marker of recurrence-free survival (RFS) and poor prognosis in hepatocellular carcinoma (HCC) [46,47]. Another study identified SOX9 as an independent risk factor for both RFS and overall survival (OS) in HCC patients treated with sorafenib, as it enhances sorafenib resistance through the modulation of ABCG2 [11]. In our experiments, we found that HCCs in the SOX9-positive group had worse outcomes than those in the SOX9-negative group when treated with sorafenib after surgery. Specifically, their RFS and OS were significantly lower. Notably, HCC recurrence post-hepatectomy is typically classified into early or late recurrence, with 12 months being the cut-off point [48]. Our results for RFS within 12 months revealed that the SOX9-positive group had a significantly lower RFS rate compared to the SOX9-negative group. We attribute this to the fact that the HCCs in the SOX9-positive group often exhibited more aggressive histological behaviors, such as stronger venous invasion and more advanced TNM stages. These findings reinforce the idea that SOX9 status serves as an independent predictor of OS and RFS in HCCs after sorafenib treatment.

The results above clearly demonstrate that the method proposed in this study can accurately identify patients with advanced hepatocellular carcinoma (HCC) who are most likely to benefit from sorafenib treatment, thus providing personalized treatment strategies, particularly in deciding whether or not to administer sorafenib. Through this approach, we are not only able to predict patients’ treatment responses based on their SOX9 status but also to tailor treatment plans to each patient to enhance treatment efficacy and reduce unnecessary drug side effects. More importantly, this personalized treatment approach has the potential to significantly improve patients’ quality of life and survival rates, ultimately optimizing treatment outcomes. This precise treatment planning can assist doctors in making better decisions during treatment, playing a crucial role in complex cancer therapies.

However, despite the promising potential of this method, it still has several limitations. First, the reinforcement learning network is relatively complex to train and prone to overfitting, especially when the sample size is small. To improve training efficiency and avoid overfitting, we introduced two different learning rates during model training, using a smaller learning rate for the RL-based network to prevent gradient explosion and ensure training stability. Second, in our experiments, the model’s specificity was always higher than its sensitivity, which could be related to the small sample size used. The insufficient sample size may have caused the model to be more conservative in avoiding false positives, resulting in higher specificity but potentially affecting its sensitivity. To overcome this limitation, future studies could increase the sample size to improve the model’s performance and further optimize its generalization ability. Additionally, clinical variables could be incorporated into the model to enhance its overall diagnostic performance, particularly when diagnosing early-stage or rare subtypes of HCC. Finally, the data in this study were derived from a single institution, and while internal validation has proven the reliability of the model, further external validation using independent cohorts from different institutions is necessary. This multi-center validation would help reduce potential biases and strengthen the model’s applicability, ensuring its operational value and utility in a broader clinical setting.

## 6. Conclusions

In this study, we developed and validated a deep learning model that incorporated reinforcement learning for the non-invasive preoperative prediction of SOX9 expression status based on CT images. Our extensive experimental results showed that our algorithm achieved AUCs of 94.42% in the training cohort and 91.00% in the validation cohort, outperforming traditional models. Additionally, our visualization experiments demonstrated that RL technology helped the model accurately identify the optimal regions for prediction. These promising results confirmed the relationship between SOX9 expression status and medical imaging and further indicated that our DL model is a promising approach for identifying SOX9 status and holds significant potential for guiding precision treatment strategies in hepatocellular carcinoma.

## Figures and Tables

**Table 1:** Comparison of the baseline characteristics of the training and validation cohorts.

Attributes	The Training Cohort		*p*-Value	The Validation Cohort		*p*-Value
	SOX9-Positive	SOX9-Negative		SOX9-Positive	SOX9-Negative	
Number	41	40	-	10	10	-
Gender			0.967			0.343
Males	36	36	-	10	8	-
Females	5	4	-	0	2	-
Age	52.07 ± 14.33	51.2 ± 11.51	0.607	52.0 ± 12.23	48.6 ± 9.30	0.840
PLT (×10^9^/L)	167.48 ± 88.16	153.54 ± 72.91	0.447	162.0 ± 67.21	160.78 ± 57.80	0.968
Neu (×10^9^/L)	5.08 ± 3.61	3.93 ± 1.57	0.073	4.29 ± 2.55	5.22 ± 2.02	0.404
Lym (×10^9^/L)	1.43 ± 0.78	1.82 ± 0.96	0.049	1.71 ± 0.59	2.22 ± 1.10	0.238
TBIL (μmol/L)	18.31 ± 10.89	18.11 ± 10.45	0.936	25.1 ± 21.70	13.99 ± 5.62	0.167
ALT (U/L)	75.56 ± 104.26	47.45 ± 37.79	0.116	121.1 ± 224.17	47.35 ± 17.00	0.351
AST (U/L)	109.66 ± 231.39	56.01 ± 53.81	0.161	71.1 ± 106.31	50.23 ± 19.47	0.570
ALB (g/L)	43.29 ± 6.38	44.70 ± 3.71	0.232	41.64 ± 5.37	44.99 ± 3.10	0.127
GGT (μ/L)	85.15 ± 57.26	85.73 ± 82.61	0.971	87.7 ± 109.42	128.69 ± 116.97	0.453
AFP (ng/mL)	493.65 ± 519.00	453.85 ± 507.72	0.849	574.53 ± 539.87	409.51 ± 461.61	0.673
CEA (ng/mL)	2.79 ± 1.16	2.27 ± 0.98	0.038	2.56 ± 1.12	3.15 ± 2.23	0.487

**Table 2:** The foundational model architecture of the proposed algorithm, including its classification model, generator, and critic.

Models	Modules	Inputs	Outputs
Classification Model	Conv2d	[− 1, 3, 128, 128]	[−1, 16, 128, 128]
	BatchNorm2d	[− 1, 16, 128, 128]	[− 1, 16, 128, 128]
	ResNet Block	[− 1, 16, 128, 128]	[− 1, 64, 128, 128]
	ResNet Block	[− 1, 64, 128, 128]	[− 1, 128, 64, 64]
	ResNet Block	[− 1, 128, 64, 64]	[− 1, 256, 32, 32]
	AdaptiveAvgPool2d	[− 1, 256, 32, 32]	[− 1, 256, 1, 1]
	Linear	([− 1, 256], [− 1, 256])	[− 1, 2]
Generator	Linear	[− 1, 256]	[− 1, 512]
	ReLu	[− 1, 512]	[− 1, 512]
	Linear	[− 1, 256]	[− 1, 512]
	ReLu	[− 1, 512]	[− 1, 512]
	Linear	[− 1, 256]	[− 1, 256]
	Tanh	[− 1, 256]	[− 1, 256]
Critic	Linear	[− 1, 256]	[− 1, 64]
	LeakyReLu	[− 1, 64]	[− 1, 64]
	Linear	[− 1, 64]	[− 1, 1]

**Table 3:** Comparison of prediction models’ performance on the training and validation cohorts in terms of accuracy (ACC) and area under the curve (AUC).

Models	The Training Cohort (N = 81)		The Validation Cohort (N = 20)	
	ACC	AUC (95%CI)	ACC	AUC (95%CI)
AlexNet [31]	61.73	61.95 (58.40–66.52)	75.00	68.00 (63.71–72.33)
MobileNetV2 [32]	67.90	68.48 (65.23–72.35)	70.00	69.00 (65.45–74.42)
DenseNet-121 [33]	77.78	82.44 (79.49–85.78)	70.00	73.00 (70.12–77.27)
InceptionV3 [30]	62.96	62.68 (58.17–67.14)	75.00	80.00 (77.32–83.15)
ResNet(baseline)	72.84	78.35 (74.16–81.82)	80.00	80.00 (75.80–83.89)
SEResNet [28]	82.72	86.04 (82.21–88.64)	80.00	72.00 (65.34–75.64)
**Ours**	**90.12**	**94.42** (92.33–96.39)	**90.00**	**91.00** (88.64–93.15)

## Data Availability

The data presented in this study are available on request from the corresponding authors.
